# BMAL1-Mediated Circadian Regulation of Oocyte Quality and IVF Outcomes

**DOI:** 10.3390/genes17080981

**Published:** 2026-08-21

**Authors:** Charalampos Voros, Nektaria Zagorianakou, Stylianos Makrydimas, Fotios Chatzinikolaou, Georgios Papadimas, Aristotelis Marios Koulakmanidis, Nikolaos Thomakos, Panagiotis Antsaklis, Georgios Daskalakis, George Makrydimas

**Affiliations:** 1 Department of Obstetrics and Gynecology, ‘Alexand’ General Hospital, National and Kapodistrian University of Athens, 80 Vasilissis Sofias Avenue, 11528 Athens, Greece; aristoteliskoulak@gmail.com (A.M.K.); thomakir@hotmail.com (N.T.); panosant@gmail.com (P.A.); gdaskalakis@yahoo.com (G.D.); 2 Scientific Laboratory for Innovative Technologies in Internal Medicine, Preventive Medicine and Overall Care, Department of Nursing, School of Health Sciences, University of Ioannina, 45500 Ioannina, Greece; zagorianakou@uoi.gr; 3 Medical School, Aristotle University of Thessaloniki, 54124 Thessaloniki, Greece; stylianosmakrydimas@hotmail.com; 4 Laboratory of Forensic Medicine and Toxicology, School of Medicine, Aristotle University of Thessaloniki, 54124 Thessaloniki, Greece; 5 Athens Medical School, National and Kapodistrian University of Athens, 15772 Athens, Greece; dr.georgepapadimas@gmail.com; 6 Department of Obstetrics & Gynecology, University Hospital of Ioannina, 45500 Ioannina, Greece; gmakrydi@uoi.gr

**Keywords:** BMAL1, circadian rhythm, circadian clock, oocyte quality, assisted reproductive technology, in vitro fertilization, granulosa cells, ovarian ageing, oxidative stress, mitochondrial dysfunction, melatonin, female infertility

## Abstract

Successful embryo development transpires far before fertilization inside the meticulously regulated microenvironment of the ovarian follicle. Increasing data indicates that circadian regulatory systems influence several processes that define oocyte competence, including mitochondrial activity, oxidative balance, meiotic development, and cellular metabolism. Brain and muscle ARNT-like protein 1 (BMAL1), an essential transcription factor in circadian regulation, has garnered significant interest due to its crucial involvement in ovarian physiology and reproductive function. Dysregulated BMAL1 signaling has been linked to compromised folliculogenesis, diminished steroidogenesis, mitochondrial dysfunction, elevated oxidative stress, and irregularities in meiotic spindle organization, all of which may negatively impact oocyte quality and early embryo development. Recent experimental and clinical findings indicate that results of assisted reproduction may be influenced by circadian disruption, sleep problems, obesity, ageing, and metabolic dysfunction.

## 1. Introduction

Circadian rhythms govern nearly every aspect of mammalian physiology, from sleep–wake cycles to hormone secretion, cell division, and metabolic function [1]. The molecular machinery driving these rhythms depends on a transcription–translation feedback loop in which the CLOCK-BMAL1 heterodimer activates the expression of Period and Cryptochrome genes via E-box promoter elements; the resulting PER-CRY complexes then accumulate and suppress CLOCK-BMAL1 activity, completing an approximately 24-h oscillation [2]. BMAL1 (Brain and Muscle ARNT-Like Protein 1) occupies a singular position within this loop: it is the only core clock component whose deletion abolishes circadian rhythmicity without compensation, and female mice lacking it are uniformly infertile [3,4].

The reproductive system is among the most temporally organized in the body. Gonadotropin release, the preovulatory LH surge, ovulation, and endometrial receptivity each depend on precise timing coordinated by the hypothalamic–pituitary–ovarian (HPO) axis [4,5]. Critically, the ovary does not simply receive circadian instructions from the suprachiasmatic nucleus (SCN); it contains autonomous peripheral clocks within granulosa cells, theca cells, and oocytes that integrate local hormonal and metabolic signals with systemic circadian input [5]. When these peripheral clocks are disrupted, the consequences extend beyond ovulation timing to steroidogenesis, oxidative stress regulation, and the developmental competence of the oocyte itself [6].

Circadian disruption has become increasingly prevalent: night shift work, irregular sleep patterns, obesity, and artificial light at night all impair clock function at the tissue level, and epidemiological data link several of these exposures to reduced female fertility and worse IVF outcomes [7,8,9,10]. Yet reproductive medicine has been slow to incorporate circadian biology into clinical practice or even into the framing of ART-related research. Our review examines the evidence on BMAL1, and specifically its roles in folliculogenesis, steroidogenesis, and oocyte quality, the consequences of its disruption across different clinical contexts, and whether any of this translates into actionable guidance for patients undergoing IVF. We focus on BMAL1 because, among the core clock components, it is the only one whose loss abolishes circadian rhythmicity without compensation by paralogous genes [3], making it the most informative single node for evaluating how clock disruption affects reproductive outcomes; other clock genes expressed in the human ovary, including PER1, PER2, CRY1, CRY2, and ARNTL2, are discussed where they intersect directly with BMAL1-dependent pathways or provide relevant comparative evidence (Section 3.1, Section 5.1 and Section 7).

## 2. Methods

A narrative review of the published literature was conducted across three databases: PubMed/MEDLINE, Scopus, and Google Scholar. Searches were conducted between March and May 2026. Searches were performed independently in each database using combinations of the following terms: “BMAL1”, “ARNTL”, “circadian clock”, “circadian rhythm”, “clock genes”, “oocyte quality”, “oocyte competence”, “IVF outcomes”, “ART outcomes”, “assisted reproductive technology”, “granulosa cells”, “theca cells”, “cumulus cells”, “steroidogenesis”, “folliculogenesis”, “ovarian follicle”, “oxidative stress”, “reactive oxygen species”, “mitochondrial function”, “meiotic spindle”, “spindle assembly”, “shift work”, “circadian disruption”, “light at night”, “sleep quality”, “melatonin”, “follicular fluid”, “ovarian aging”, “female infertility”, and “obesity infertility”. Boolean operators (AND, OR) were used to combine terms across categories; for example, (“BMAL1” OR “ARNTL” OR “circadian clock”) AND (“oocyte” OR “folliculogenesis” OR “steroidogenesis”) AND (“IVF” OR “ART” OR “fertility”).

No formal date restriction was applied at the initial search stage. Given the relatively recent characterization of peripheral clock function in reproductive tissues, the majority of relevant literature falls within 2010–2025, and this range was used as a practical filter during title and abstract screening. Reference lists of retrieved articles were also hand-searched to identify additional studies not captured by the database searches. Records retrieved from the three databases were compared and duplicate citations were removed before screening. The literature search, including screening of retrieved records for eligibility, was performed jointly by two authors (C.V. and N.Z.).

Articles were considered eligible if they met at least one of the following criteria: (i) they reported on BMAL1 or other core clock gene expression or function in ovarian or reproductive tissues, in either animal models or humans; (ii) they examined the relationship between circadian disruption from any cause and fertility, ovarian function, or ART outcomes; (iii) they investigated the role of melatonin in oocyte quality, granulosa cell function, or IVF outcomes; or (iv) they addressed obesity- or aging-related changes in circadian clock function with reproductive implications. Both original research articles and previously published reviews were included where relevant. Studies not available in English, conference abstracts without full-text publications, and articles with confirmed retraction notices were excluded. Given the narrative format of this review, no formal quality scoring or risk-of-bias assessment was applied. However, study design, sample size, and species of origin were considered when weighting the evidence, and mechanistic findings from rodent models are explicitly distinguished from human clinical data throughout the text. A representative search combination (BMAL1 AND oocyte AND fertility) currently returns 158 records in PubMed/Europe PMC. As this is a narrative rather than a systematic review, records were not tracked through a formal PRISMA-style flow [11]; the final synthesis draws on 74 primary research and review articles.

**Figure 1:**
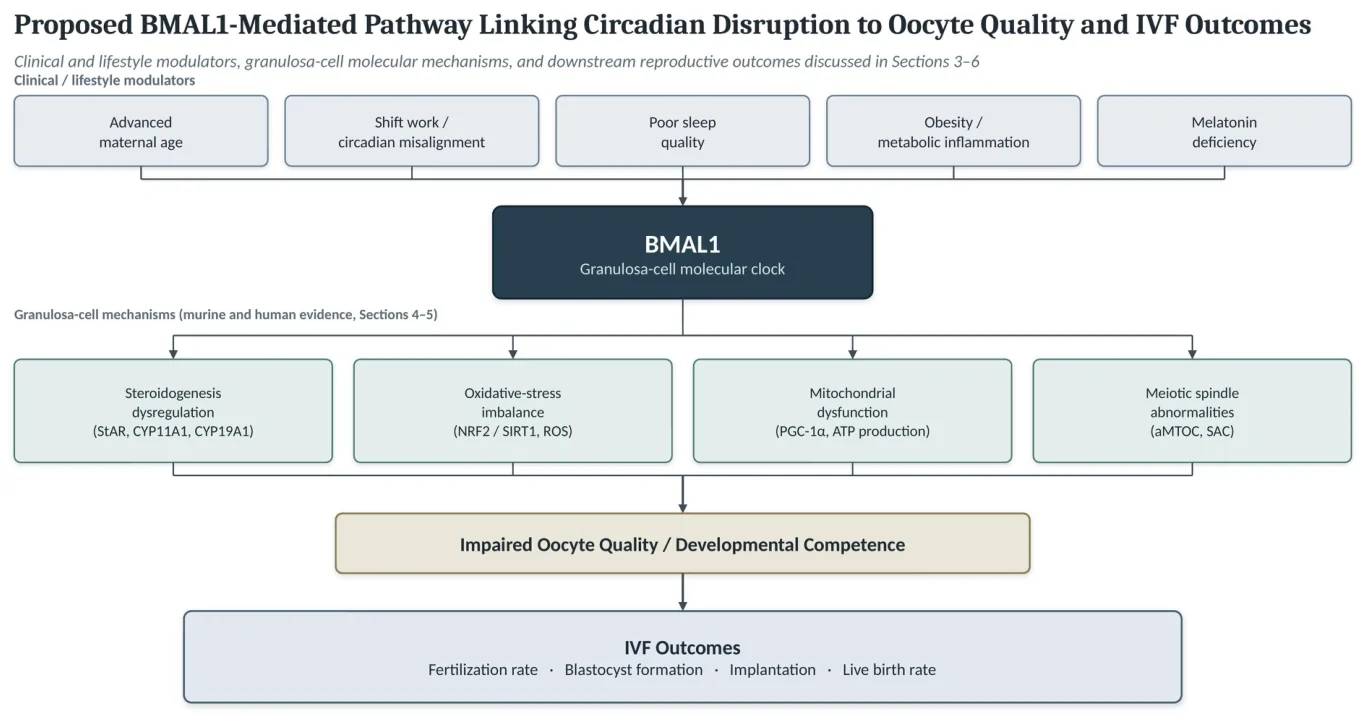
Proposed schematic linking circadian and lifestyle modulators to granulosa-cell BMAL1 signaling, downstream cellular mechanisms, oocyte quality, and IVF outcomes. Arrows indicate mechanistic associations reported in the murine and human granulosa-cell literature discussed in Section 3, Section 4, Section 5 and Section 6; the scheme is a proposed conceptual model and does not represent an established clinical algorithm.

To orient the mechanistic and clinical evidence reviewed in the following sections, Figure 1 summarizes the proposed pathway linking clinical and lifestyle circadian disruptors to granulosa-cell BMAL1 signaling, the downstream cellular mechanisms discussed in Section 3, Section 4, Section 5 and Section 6, and oocyte quality and IVF outcomes.

## 3. BMAL1 in Ovarian Biology: From Follicle Development to Steroidogenesis

### 3.1. The Molecular Clock in Ovarian Cells

The mammalian circadian clock is built on two interlocking transcriptional feedback loops. In the primary loop, the CLOCK-BMAL1 heterodimer binds E-box elements (CACGTG) in the promoters of target genes and drives the rhythmic transcription of Period (Per1, Per2) and Cryptochrome (Cry1, Cry2) genes [12]. As PER and CRY proteins accumulate in the cytoplasm, they form repressor complexes, translocate to the nucleus, and directly inhibit CLOCK-BMAL1 transcriptional activity, thereby suppressing their own synthesis [13]. The gradual degradation of these repressor complexes mediated largely by casein kinase 1δ/ε-dependent phosphorylation and subsequent proteasomal clearance releases CLOCK-BMAL1 from inhibition and allows the next transcriptional cycle to begin, completing an oscillation of approximately 24 h [2]. A secondary stabilizing loop operates in parallel: the nuclear receptors REV-ERBα and REV-ERBβ, themselves transcriptional targets of CLOCK-BMAL1, repress Bmal1 transcription by binding RORE elements in its promoter, while the competing ROR receptors (RORα, RORγ) activate it. The balance between REV-ERB and ROR activity fine-tunes the amplitude and phase of BMAL1 expression and, by extension, the robustness of the entire oscillator [2,4].

Within this architecture, BMAL1 occupies a uniquely indispensable position. Unlike PER and CRY proteins, which exist as multiple partially redundant paralogs, BMAL1 has no functional substitute at the level of the primary loop its deletion produces arrhythmicity that cannot be compensated by BMAL2 or any other family member under physiological conditions [3]. This non-redundancy explains why global Bmal1 knockout produces a more severe reproductive phenotype than deletion of individual Per or Cry genes, and why BMAL1 has attracted particular attention as a potential mediator of clock-related pathology [14].

Beyond timekeeping, BMAL1 functions as a broad transcriptional regulator with thousands of genomic binding sites that extend well beyond canonical clock output genes. Genome-wide ChIP-seq studies have shown that BMAL1 occupancy is highly tissue-specific, shaped by the chromatin accessibility and co-factor landscape of each cell type [15]. In the ovary, this means that the BMAL1 cistrome in granulosa cells is distinct from that in theca cells, oocytes, or any somatic tissue, and that the downstream consequences of BMAL1 loss differ accordingly across follicular compartments. Among the genes directly regulated by BMAL1 in steroidogenic cells are those encoding enzymes and transport proteins in the cholesterol-to-steroid biosynthesis pathway, including Star, Cyp11a1, Hsd3b2, and Cyp19a1 genes whose products catalyze sequential steps from mitochondrial cholesterol import through to estrogen synthesis [16,17]. The E-box mediated regulation of these genes means that their expression oscillates with the circadian cycle, peaking at specific phases that are presumably aligned with the timing of gonadotropin stimulation and ovulation under physiological conditions.

The ovary expresses clock genes not only in somatic follicular cells but also in the oocyte itself, though the functional significance of the oocyte clock remains less well characterized than that of granulosa or theca cells [18]. In granulosa cells, circadian gene expression has been confirmed in both rodent and human tissue, with PER2 showing the most consistent oscillatory pattern in human luteinized granulosa cells, while CLOCK and BMAL1 display lower amplitude rhythms that may reflect the luteinization process or the absence of synchronizing cues in isolated cell preparations [16,17]. The corpus luteum, which forms from the ruptured follicle after ovulation, also expresses clock genes, and BMAL1 protein levels in luteal tissue increase substantially during corpus luteum formation a pattern consistent with an active role in supporting the progesterone output required for early pregnancy maintenance [5].

The ovarian clock does not simply receive instructions from the SCN. Several lines of evidence indicate that it can sustain autonomous oscillations in explanted ovarian tissue and in isolated granulosa cells following synchronization stimuli such as dexamethasone or forskolin [5,17]. This peripheral autonomy has functional consequences: the ovarian clock integrates local signals including follicle-stimulating hormone (FSH), luteinizing hormone (LH), insulin, and metabolic substrates that can reset or entrain its phase independently of central clock input. LH, in particular, has been shown in rodent studies to acutely induce Per1 expression in granulosa cells, effectively using the clock machinery as part of the cellular response to the ovulatory trigger [5]; whether this LH-Per1 response is conserved in human granulosa cells has not been directly tested. This bidirectional relationship between gonadotropin signaling and clock gene expression means that disruption at either level impaired LH signaling or impaired BMAL1 function can propagate dysfunction through the other pathway, amplifying the reproductive deficit [19].

The tissue-specific and signal-responsive nature of BMAL1 function in the ovary has a direct implication for how circadian disruption should be understood in the reproductive context. Systemic markers of circadian misalignment such as dim-light melatonin onset, actigraphy-derived sleep timing, or peripheral blood clock gene expression may not accurately reflect the functional state of the ovarian clock, particularly in patients whose disruption originates from metabolic or local inflammatory signals rather than from altered light exposure [20]. This disconnect between systemic and tissue-level clock function is a recurring limitation in translating animal clock biology to clinical reproductive medicine, and it underscores the need for follicular fluid or granulosa cell-based assessments of clock function in future clinical studies.

### 3.2. Folliculogenesis and Ovulation Timing

Folliculogenesis is a protracted process spanning several months in humans, during which primordial follicles are recruited, grow through preantral and antral stages, and ultimately either reach ovulation or undergo atresia. For most of this period, follicular development proceeds independently of gonadotropin stimulation, driven instead by intraovarian paracrine signals and growth factors [21]. Clock gene expression has been documented across these early stages, suggesting that the circadian machinery is active throughout follicular life and not merely at the point of ovulatory maturation [4]. The functional relevance of this early clock activity is not yet fully resolved, but given that BMAL1 regulates genes controlling cell cycle progression, apoptosis, and metabolic substrate utilization, its expression during the extended preantral phase likely influences the trajectory of follicle development long before gonadotropin dependence is established [22].

As follicles enter the gonadotropin-dependent phase, the relationship between clock function and reproductive signaling becomes more direct. FSH receptor expression in granulosa cells, and the downstream cAMP signaling cascade it initiates, are subject to circadian modulation in rodent models FSH-induced cAMP accumulation varies with the phase of the granulosa cell clock, meaning that the steroidogenic and proliferative response to a given FSH stimulus is not constant across the 24-h cycle [5]; whether this holds in human granulosa cells is untested. Whether this pattern implies that timing gonadotropin administration to follicular clock phase could improve controlled ovarian stimulation in IVF is a hypothesis, not an established clinical implication: exogenous gonadotropin administration is currently timed according to clinical convenience rather than follicular clock phase, and whether stimulation at different times of day produces systematically different follicular responses has not been formally studied [23] (see Section 7 for why this remains untested in the clinic).

The transition from FSH dependence to LH dependence at the preovulatory stage introduces a second clock-regulated checkpoint. The LH surge in rodents occurs in a narrow circadian window gated by the SCN through vasoactive intestinal peptide (VIP) projections to GnRH neurons, and disrupting this gating mechanism by lesioning the SCN, altering the light cycle, or deleting Bmal1 delays or abolishes the surge [24]. In humans, where the LH surge is less rigidly phase-locked to the light cycle, the circadian regulation of GnRH pulsatility is less well characterized, but there is evidence that LH secretion retains diurnal variation and that this variation is disrupted in women with irregular sleep patterns or shift work exposure [7,8]. In the context of natural conception, a mistimed LH surge relative to the window of peak oocyte maturation would result in ovulation of a suboptimally prepared oocyte. In IVF, where the trigger injection replaces the endogenous LH surge, this particular vulnerability is bypassed but clock-dependent processes operating downstream of the surge, including cumulus expansion, meiotic resumption, and cortical granule redistribution, would be expected to remain subject to intrinsic follicular clock regulation, although this has not been directly tested in IVF patients [25].

Xu et al. provided the most quantitatively detailed characterization of the folliculogenesis phenotype in Bmal1-null females [26]. In addition to the reduction in naturally ovulated oocyte numbers and the increase in morphological abnormalities after superovulation already noted, they documented impaired blastocyst formation rates from fertilized Bmal1^−/−^ oocytes (*p* < 0.01) and a significant reduction in implantation sites per female (3.4 ± 0.5 vs. 6.9 ± 0.7; *p* < 0.001) when Bmal1^−/−^ oocytes were transferred into wild-type recipients. This last finding is particularly informative: by controlling for uterine environment through embryo transfer into wild-type recipients, the study isolated the oocyte and early embryo as the locus of the developmental deficit. The problem is not an inhospitable uterus it is an oocyte that lacks the developmental reserves to sustain embryo progression through implantation, regardless of the quality of the environment it enters.

Cumulus cells, which surround the oocyte and provide metabolic and paracrine support during maturation, also express clock genes, and BMAL1 in cumulus cells has been proposed to coordinate the timing of gap junction closure, hyaluronan synthesis during cumulus expansion, and the shift in energy substrate utilization from oxidative phosphorylation to glycolysis that accompanies final oocyte maturation [5]. Disruption of cumulus cell clock function could therefore impair oocyte maturation support independently of any intrinsic oocyte clock deficit, adding a further layer through which BMAL1 dysfunction compromises the oocyte developmental program. Whether cumulus cell clock gene expression in IVF patients correlates with maturation rates or embryo quality is an open question that could, in principle, be addressed using existing biobank samples from ART programs [6].

### 3.3. Steroidogenesis: The StAR-Progesterone Axis

Steroid hormone synthesis in the ovary follows a compartmentalized two-cell model in which theca cells produce androgens under LH stimulation, and granulosa cells convert those androgens to estrogens via aromatase (CYP19A1) under FSH control. After ovulation, the granulosa and theca cells of the ruptured follicle luteinize and redirect their steroidogenic output toward progesterone, which is essential for endometrial preparation and early pregnancy maintenance [27]. Each enzymatic step in this pathway from mitochondrial cholesterol import through to estrogen and progesterone secretion depends on a series of tightly regulated proteins whose expression, as it turns out, is subject to direct circadian control via BMAL1 [16,17,28].

The rate-limiting step in steroidogenesis is not enzymatic but physical: the transport of cholesterol from the outer to the inner mitochondrial membrane, a process mediated by the Steroidogenic Acute Regulatory protein (StAR) [27]. Without adequate StAR activity, cholesterol cannot reach CYP11A1 (the side-chain cleavage enzyme) on the inner membrane, and the entire downstream cascade stalls regardless of how much substrate is available or how active the downstream enzymes are. StAR expression is acutely regulated by LH signaling through cAMP-dependent mechanisms, but it is also under circadian transcriptional control: its promoter contains functional E-box elements that are occupied by BMAL1-CLOCK in a phase-dependent manner, driving rhythmic StAR mRNA accumulation in luteinized follicle cells in mice [26]. This dual regulation acute hormonal and tonic circadian positions StAR as a node where disruption of either signaling axis converges to produce the same downstream deficit in steroid output.

Liu et al. demonstrated this convergence genetically [16]. In SF1-Bmal1^−/−^ females, where BMAL1 is absent specifically from steroidogenic cells while the rest of the clock network remains intact, circulating progesterone levels on day 3.5 of pseudopregnancy were significantly lower than in controls (1.8 ± 0.4 vs. 8.2 ± 1.3 ng/mL; *p* < 0.001), and uterine implantation sites were absent. Microarray analysis of knockout corpora lutea identified StAR as among the most substantially downregulated transcripts, alongside Aldob and a cluster of metabolic genes involved in mitochondrial substrate handling. The specificity of the conditional deletion to steroidogenic cells meant that the HPO axis, endometrial receptivity, and embryo quality were unaffected the entire phenotype was attributable to insufficient luteal progesterone production stemming from loss of BMAL1-driven StAR transcription.

Downstream of StAR, CYP11A1 converts cholesterol to pregnenolone, which is then processed by HSD3B2 to progesterone, or shunted toward the androgen pathway via CYP17A1 in theca cells. Each of these enzymes shows circadian variation in expression in steroidogenic tissues, and several carry functional E-box elements in their promoters [17,28]. Wang et al. mapped this across multiple steroidogenic genes simultaneously in Bmal1-knockdown mouse granulosa and theca cells, documenting suppressed mRNA levels of Star, Hsd3b2, Cyp19a1, and Lhcgr in parallel with reduced androgen and progesterone concentrations in culture supernatants [28]. The simultaneous downregulation of Lhcgr encoding the LH receptor is noteworthy because it implies that BMAL1 loss does not merely reduce the capacity for steroid synthesis but also blunts the sensitivity of granulosa cells to the LH signal that drives luteinization in the first place. Loss of clock function therefore impairs steroidogenesis at two distinct levels: transcriptional control of biosynthetic enzymes and receptor-level responsiveness to the gonadotropin trigger.

Wang et al. also observed that Bmal1 knockdown activated PI3K/NF-κB signaling in mouse luteinized granulosa cells. This is mechanistically significant beyond its immediate steroidogenic implications [28]. NF-κB is a master regulator of inflammatory gene expression, and its activation in granulosa cells promotes the production of pro-inflammatory cytokines including IL-6 and TNF-α that directly suppress steroidogenic enzyme expression and accelerate granulosa cell apoptosis [29]. The NF-κB-BMAL1 relationship is not unidirectional: Maury et al. subsequently demonstrated in human adipose tissue that NF-κB physically competes with BMAL1 for occupancy at shared genomic loci, effectively displacing the clock machinery from its target sites under inflammatory conditions [15]. Whether the same competitive displacement occurs in ovarian granulosa cells under conditions of clock disruption has not been directly tested, but the convergence of these findings suggests a self-reinforcing cycle in which BMAL1 loss activates NF-κB, which further suppresses BMAL1 target gene expression and clock amplitude, progressively eroding steroidogenic capacity [6].

The human data provided by Kawamura et al. ground these mechanistic findings in clinical reality [17]. Luteinized granulosa cells collected from IVF patients at oocyte retrieval showed a significant positive correlation between BMAL1 mRNA levels and those of CYP11A1, CYP19A1, STAR, and ESR2 across the patient cohort, with higher BMAL1 expression predicting greater steroidogenic gene activity at the individual level [17]. Functional experiments in KGN and HGL5 cell lines confirmed causality in both directions: BMAL1 overexpression increased CYP19A1 mRNA and 17β-estradiol secretion, while siRNA-mediated knockdown reduced them. These human cell line data, taken together with the patient cohort correlations, establish that BMAL1 is not merely associated with steroidogenic capacity in the human follicle but actively regulates it and raise the speculative possibility, not yet tested against implantation outcomes, that the degree of BMAL1 expression in granulosa cells at the time of oocyte retrieval could serve as a patient-specific marker of luteal phase adequacy; whether it predicts implantation success has not been examined [17]. The steroidogenic targets of BMAL1 identified across experimental and human studies are summarized in Table 1.

## 4. BMAL1, Oocyte Quality, and the Redox-Mitochondrial Axis

### 4.1. Oxidative Stress and the Follicular Redox Environment

Reactive oxygen species are produced continuously within the follicle as byproducts of mitochondrial oxidative phosphorylation, cytochrome P450-mediated steroidogenesis, and NADPH oxidase activity in granulosa cells [30]. At physiological concentrations, ROS serve as intracellular signaling molecules that participate in the regulation of meiotic resumption, cumulus expansion, and ovulation processes that are themselves temporally coordinated and require precise redox conditions at specific developmental stages [31]. The granulosa cell layer surrounding the oocyte is the primary source of follicular fluid antioxidants, secreting reduced glutathione, ascorbic acid, and catalase into the follicular microenvironment at concentrations that exceed those in peripheral blood, reflecting the degree to which the follicle actively maintains a controlled redox state rather than simply equilibrating with systemic oxidative conditions [32]. This local antioxidant investment is not uniform across follicle size or developmental stage: antioxidant enzyme activity rises as follicles mature, peaking in the preovulatory period when ROS production from steroidogenesis and prostaglandin synthesis is at its highest, and the adequacy of this response at the critical maturation window is a determinant of whether the oocyte exits meiosis I with intact chromosomal architecture or accumulates irreversible oxidative damage [33].

The problem arises when ROS production exceeds the buffering capacity of the follicular antioxidant system, shifting the redox balance toward oxidative stress. In that state, ROS attack mitochondrial DNA, which lacks the histone protection and nucleotide excision repair machinery available to nuclear DNA and is therefore disproportionately vulnerable to oxidative base modifications [34]. Oxidized mitochondrial DNA impairs the transcription of mitochondrially encoded electron transport chain subunits, reducing respiratory capacity in a self-amplifying cycle that is particularly damaging in the oocyte, where mitochondrial function cannot be replenished by fusion with a healthy mitochondrial network as it can in somatic cells [30,31]. Beyond mitochondrial DNA, ROS oxidize lipid membranes particularly the polyunsaturated fatty acid-rich membranes of mitochondria and the endoplasmic reticulum generating lipid peroxidation products such as 4-hydroxynonenal that form adducts with spindle proteins, carbonylate cytoskeletal components, and activate intrinsic apoptotic pathways in granulosa cells [35]. This cascade of oxidative injury, once initiated in the preovulatory follicle, is largely irreversible by the time the oocyte reaches metaphase II, and the damage it causes is not corrected during fertilization or early cleavage, propagating instead into the embryo where it manifests as developmental arrest, fragmentation, or aneuploidy.

BMAL1 sits upstream of several antioxidant defense mechanisms that normally prevent this shift. At the transcriptional level, BMAL1-CLOCK complexes drive the rhythmic expression of Nrf2, the master regulator of the cellular antioxidant response, and of Sirt1, a NAD+-dependent deacetylase that stabilizes NRF2 protein and suppresses NF-κB-driven pro-oxidant gene programs [4,30]. NRF2 activation requires its release from the KEAP1 ubiquitin ligase complex, which constitutively targets NRF2 for proteasomal degradation under basal conditions; oxidative or electrophilic stress modifies critical cysteine residues on KEAP1, releasing NRF2 to translocate to the nucleus and activate antioxidant response element (ARE)-driven genes [36]. SIRT1 facilitates this process by deacetylating NRF2 at lysine residues that would otherwise promote its nuclear export, effectively prolonging NRF2 transcriptional activity in response to oxidative challenge [30]. The downstream targets of NRF2 in granulosa cells include superoxide dismutase 2 (SOD2), which dismutates mitochondrial superoxide to hydrogen peroxide; glutathione peroxidase (GPx1, GPx4), which reduces hydrogen peroxide and lipid hydroperoxides using glutathione as the electron donor; heme oxygenase-1 (HO-1), which catabolizes pro-oxidant free heme and generates the antioxidant biliverdin; and thioredoxin reductase, which regenerates the thioredoxin system responsible for reducing oxidized peroxiredoxins [4,30,31]. The circadian gating of this entire response means that antioxidant capacity in the follicle is not static but oscillates in phase with metabolic activity and ROS production, providing heightened protection during periods of peak oxidative load. When BMAL1 function is lost, this oscillation collapses, NRF2 activity falls across the entire cycle rather than at discrete phases, and the follicle’s capacity to buffer oxidative insults is chronically reduced rather than transiently overwhelmed [37].

Xu et al. measured the consequences of this collapse directly in Bmal1^−/−^ mice, finding significantly elevated intracellular ROS in ovarian tissue on proestrus and in oviductal tissue on metestrus relative to wild-type controls [26]. The temporal specificity of these measurements is informative: elevated ROS on proestrus coincides with the period of final oocyte maturation and the preovulatory LH surge, when steroidogenesis-driven ROS production peaks and antioxidant demand is highest, while elevated oviductal ROS on metestrus corresponds to the transit of the fertilized oocyte toward the uterus two developmental windows in which oxidative damage would be expected to have maximal consequences for fertilization efficiency and early embryo survival. The follicular fluid of Bmal1 -/- females also showed reduced concentrations of glutathione and ascorbate relative to controls, indicating that the elevated ROS reflects not only increased production but also depleted scavenging capacity a combination that is more damaging than either perturbation alone [26]. Whether this pattern parallels the oxidative profile reported in follicular fluid from older IVF patients or women with unexplained infertility has not been directly tested and remains a hypothesis to be confirmed in human samples rather than an established finding. The correlation between these ROS elevations and the downstream deficits in fertilization rate, blastocyst formation, and implantation potential observed in the same animals is consistent with causality, though definitive rescue experiments using targeted antioxidant delivery to the follicle would be needed to establish the relationship unambiguously.

Voros et al. elaborated the mechanistic interface between circadian oscillations and redox cycling in the oocyte specifically, proposing that the 24-h redox rhythm in which the ratio of oxidized to reduced glutathione, NADH/NAD+ balance, and peroxiredoxin oxidation state all oscillate in a clock-dependent manner functions as a secondary timing signal that coordinates meiotic progression with the metabolic state of the cell [31]. Peroxiredoxins are a family of thiol-dependent peroxidases that undergo rhythmic hyperoxidation and reduction through a mechanism that is independent of transcription and persists in cells treated with transcriptional inhibitors, in anucleate red blood cells, and even in isolated organelles establishing them as a non-transcriptional circadian oscillator that operates in parallel with the canonical BMAL1-driven feedback loop [38]. In the oocyte, which undergoes global transcriptional silencing during meiotic maturation as chromatin condenses around the germinal vesicle and the nuclear envelope breaks down, this peroxiredoxin-based redox oscillation may represent the primary mechanism through which temporal coordination is maintained during the hours between germinal vesicle breakdown and metaphase II arrest [39]. The transition through these stages germinal vesicle breakdown, metaphase I, first polar body extrusion, and metaphase II arrest follows a stereotyped timeline that in vivo is synchronized with the circadian-gated LH surge, and experimental evidence suggests that disrupting the cellular redox state at specific points within this timeline, rather than globally, is sufficient to arrest meiosis or produce chromosome segregation errors [40]. Disruption of BMAL1 in surrounding granulosa cells would be expected to desynchronize the oocyte-intrinsic redox oscillation from the metabolic and paracrine support provided by the cumulus-oocyte complex. Introducing a temporal mismatch between the oocyte’s internal developmental clock and the external signals coordinating its maturation a form of intraovarian circadian misalignment that is distinct from, and potentially additive with, the systemic circadian disruption caused by shift work or sleep disturbance [6].

### 4.2. Mitochondrial Function and Energy Supply

The mature oocyte contains between 100,000 and 500,000 mitochondria. No other cell type accumulates this many, and the reason is straightforward: from meiotic resumption through to zygotic genome activation at the 4–8 cell stage in humans, the embryo cannot synthesize new mitochondria or import them from outside [41]. Every ATP molecule required for chromosome segregation, cytoskeletal reorganization, compaction, and blastocoel formation must come from mitochondria already present in the egg at fertilization [4,30]. This makes oocyte mitochondrial quality a fixed endowment whose adequacy is determined during oogenesis, not correctable afterward. Measured by membrane potential, ATP content, and mtDNA copy number, oocyte mitochondrial quality declines with age and predicts fertilization rate, blastocyst formation, and clinical pregnancy rate in IVF with reasonable consistency across studies [30].

BMAL1 regulates mitochondrial biogenesis through PGC-1α, the master coactivator that drives mitochondrial mass expansion and oxidative capacity. PGC-1α does not bind DNA directly it coactivates NRF1, TFAM, and ERRα to coordinately induce the nuclear-encoded subunits of the respiratory chain and drive replication of the mitochondrial genome [4,30]. Its expression oscillates in a BMAL1-dependent manner, peaking during the phase of maximal oxidative metabolism. When BMAL1 is absent, PGC-1α rhythmicity dampens and mean expression falls. The resulting mitochondria are fewer, smaller, show abnormal cristae morphology, and generate less ATP per unit of substrate, based on evidence from other BMAL1-deficient tissues rather than granulosa cells specifically [42]. They also leak more electrons at complexes I and III, generating superoxide rather than completing the full reduction of oxygen to water. In granulosa cells, this reduced mitochondrial capacity compounds the StAR-mediated steroidogenic deficit already described in Section 3.3 steroidogenesis requires both the substrate (cholesterol) and the reducing equivalents and acetyl-CoA that functional mitochondria supply, so clock-driven mitochondrial impairment strikes the same pathway at a second point [43].

SIRT1 connects mitochondrial integrity to apoptotic control through FOXO3a deacetylation, which promotes transcription of MnSOD and catalase and suppresses mitochondrial permeability transition pore opening, a pathway demonstrated in other cell types rather than granulosa cells specifically [44]. When BMAL1 fails to sustain SIRT1 expression, pore opening threshold falls, inner membrane depolarization follows, and cytochrome c release initiates caspase-mediated apoptosis. Granulosa cell apoptosis is the principal driver of follicular atresia, and the clock-dependent regulation of this apoptotic threshold may influence not only individual follicle fate but also the rate of ovarian reserve depletion over time. Whether chronic circadian disruption accelerates follicle loss through this mechanism independently of age has not been directly tested in humans, but the biological plausibility is substantial and the question has clear clinical relevance for women in occupations involving long-term shift work exposure [45].

### 4.3. Meiotic Spindle Assembly and Chromosomal Segregation

The oocyte assembles its meiotic spindle without centrioles. Instead of the canonical centrosome-based nucleation used in somatic cells, it relies on acentriolar microtubule organizing centers (aMTOCs) that form from a cytoplasmic pool of pericentrin, γ-tubulin, and associated proteins, coalesce into two organized foci, and bipolarize to generate the barrel-shaped spindle that captures and segregates chromosomes [4,26,31]. This process is energy-intensive and redox-sensitive at every step. aMTOC formation requires Aurora A kinase activity, which depends on both ATP availability and the absence of oxidative inactivation of its catalytic cysteine. Spindle bipolarization requires PLK1 and CDK1, whose activities are similarly sensitive to the cellular energy and redox state. Microtubule dynamics the continuous polymerization and depolymerization that allow the spindle to capture kinetochores and generate tension depend on GTP hydrolysis and on tubulin dimers that have not been oxidatively carbonylated or cross-linked by lipid peroxidation products [4,31].

BMAL1 dysfunction impairs each of these dependencies in parallel. Reduced mitochondrial ATP output limits the energy available for kinase activity and motor protein function. Elevated ROS would be expected to oxidize tubulin and spindle-associated proteins, altering their polymerization kinetics and reducing the dynamic instability required for correct kinetochore capture, by analogy with redox-sensitive mitochondrial and cytoskeletal effects documented in other BMAL1-deficient mouse tissues [46]; this has not been directly demonstrated in oocytes. Downstream suppression of CDK1 by oxidative stress delays or prevents the metaphase I to metaphase II transition. The combined effect is a spindle that is disorganized, elongated, or geometrically asymmetric, producing impaired tension across sister kinetochores and triggering sustained activation of the spindle assembly checkpoint (SAC) [26,31]. If the SAC arrests meiosis, the oocyte fails to complete maturation. If it is overridden as occurs more frequently in older oocytes with weakened checkpoint stringency chromosomes segregate unequally, producing an aneuploid egg.

Aneuploidy in the oocyte is the most common cause of failed fertilization, embryo arrest, implantation failure, and miscarriage in IVF, and its incidence rises steeply with female age. That rise has traditionally been attributed to intrinsic oocyte aging deterioration of cohesins holding bivalent chromosomes together, accumulated DNA damage, and declining SAC stringency over decades [47]. The data reviewed here add a granulosa cell dimension to this picture. If aging reduces BMAL1 expression in granulosa cells, as Brzezinski et al. documented, and if that reduction impairs the mitochondrial and antioxidant support provided to the oocyte during maturation, then the age-related rise in aneuploidy is not solely an intrinsic oocyte phenomenon it is partly a failure of the follicular microenvironment to sustain the energetic and redox conditions that faithful chromosome segregation requires [48]. The two mechanisms are not mutually exclusive; they are more likely additive, which would explain why the aneuploidy rate accelerates disproportionately after age 37–38 rather than increasing linearly from early reproductive life.

## 5. Circadian Disruption in Clinical Context: Aging, Shift Work, and Sleep

Section 3 and Section 4 established the molecular mechanisms through which BMAL1 governs granulosa cell steroidogenesis, redox balance, mitochondrial function, and meiotic competence. The following two sections examine the clinical and lifestyle contexts in which these BMAL1-dependent processes are most plausibly disrupted in practice. The strength of the evidence linking each exposure to BMAL1 specifically, rather than to circadian disruption in general, varies: ovarian aging (Section 5.1) and obesity (Section 6.2) are supported by studies that measure BMAL1 expression or BMAL1-dependent signaling directly, whereas the shift-work and sleep literature (Section 5.2 and Section 5.3) is drawn primarily from epidemiological and hormonal data in which BMAL1 itself has not been directly assayed; where this is the case, the connection to the BMAL1 mechanisms described in Section 3 and Section 4 is stated as a hypothesis rather than an established finding.

### 5.1. Ovarian Aging and Clock Gene Decline

Ovarian aging is conventionally framed in terms of follicle number the progressive depletion of the primordial follicle pool from a peak of approximately 6–7 million at mid-gestation to fewer than 1000 at menopause. This quantitative framing, while accurate, leaves unexplained a well-documented qualitative dimension: oocyte competence declines with age at a rate that outpaces follicle loss, and the mechanisms driving this qualitative deterioration are only partially understood [49]. Chromosomal cohesion loss, accumulated mitochondrial DNA mutations, and declining SAC stringency have all been implicated, but none fully accounts for the steep acceleration in aneuploidy and implantation failure observed after the mid-thirties. The circadian clock in granulosa cells represents an underexplored contributor to this picture.

Brzezinski et al. measured the expression of eight circadian genes CRY1, CRY2, PER1, PER2, CLOCK, ARNTL/BMAL1, ARNTL2, and NPAS2 in human luteinized granulosa cells collected at oocyte retrieval from two age groups: 18–33 years and 39–45 years [48]. A consistent downward trend in clock gene expression was observed with advancing age across nearly all genes measured, reaching statistical significance for PER1 and CLOCK. The pattern was not limited to a single component but reflected a broad attenuation of the entire oscillator, suggesting that what deteriorates with age is not one clock gene in isolation but the coherence and amplitude of the granulosa cell clock as a whole. This distinction matters: a clock with reduced amplitude does not simply tick more slowly it loses the sharpness of its transcriptional peaks and troughs, flattening the rhythmic output of all downstream target genes, including those controlling steroidogenesis, antioxidant defense, and mitochondrial biogenesis discussed in previous sections [50].

The functional consequences of this clock attenuation in older granulosa cells have not been directly measured in the same patient cohorts, which is a significant gap in the current evidence. However, the mechanistic connections are sufficiently well established to make the inference reasonable: if BMAL1 amplitude declines with age in granulosa cells, StAR expression would be expected to be less robustly driven, NRF2-dependent antioxidant capacity would be expected to be chronically reduced, PGC-1α-mediated mitochondrial biogenesis would be expected to fall, and the follicular microenvironment would be expected to provide progressively less adequate support for oocyte maturation a hypothesis that has not yet been tested directly [30]. These are precisely the deficits that characterize the follicular fluid and granulosa cell phenotype of older IVF patients in published transcriptomic and metabolomic analyses, though none of those studies were designed to test circadian gene expression as a primary variable [17,48]. The convergence of findings from different methodological approaches points consistently in the same direction, even if the causal chain has not been experimentally closed in humans.

One mechanistic link between aging and clock gene attenuation that deserves attention is the progressive decline in NAD+ availability with age. NAD+ is an obligate cofactor for SIRT1, which in turn deacetylates and activates BMAL1 post-translationally, stabilizing the core clock feedback look. As NAD+ levels fall with age driven by declining NAMPT activity, the rate-limiting enzyme in the NAD+ salvage pathway SIRT1 activity falls proportionally, BMAL1 stability decreases, and clock amplitude is attenuated [51]. This represents a direct biochemical mechanism linking the metabolic aging of granulosa cells to deterioration of their circadian oscillator, and it is potentially reversible: NAD+ precursor supplementation with NMN or NR has been shown to restore SIRT1 activity and improve oocyte quality in aged mouse models, though whether this effect operates through clock restoration specifically has not been established [30]. The possibility that NAD+ precursor supplementation improves IVF outcomes in older patients through a clock-mediated mechanism is speculative at present but biologically coherent and worth investigating.

### 5.2. Shift Work and Circadian Misalignment

Night shift work and rotating schedules are among the most prevalent forms of circadian disruption in industrialized populations, affecting an estimated 15–20% of the working-age workforce in Europe and North America. For women of reproductive age, reproductive associations with this exposure have been documented across multiple study designs and outcome measures; as none of these designs are randomized, causality cannot be established from this literature alone [52]. Menstrual cycle irregularities, including cycle length variability and increased prevalence of short cycles, are consistently more common in shift workers than in day workers matched for age and BMI [7,8]. Conception rates are lower, time-to-pregnancy is longer, and rates of spontaneous abortion are elevated findings that have been replicated in prospective cohort studies controlling for confounders including physical activity, caffeine intake, and socioeconomic status [8].

Murphy and Sellix synthesized the mechanistic basis for these associations by comparing human epidemiological data with rodent models of light-cycle perturbation [5,53]. The central finding across both contexts was that repeated circadian misalignment whether from rotating shifts, phase-advanced or phase-delayed light schedules, or constant light exposure disrupts the temporal coordination of GnRH pulsatility, LH surge timing, and ovarian clock gene expression in ways that cannot be compensated by the HPO axis. In rodents, the circadian gate on the LH surge is particularly vulnerable: even a single 6-h phase shift of the light dark cycle, equivalent to transmeridian travel across six time zones, is sufficient to delay or suppress the preovulatory LH surge and prevent ovulation in that cycle [53]. The recovery of normal surge timing after phase shifting takes several cycles, during which the ovarian peripheral clock and the SCN-driven central signal are misaligned a period of internal desynchrony during which follicles continue to develop under gonadotropin stimulation but the ovulatory trigger is mistimed relative to the window of peak oocyte maturation [54].

In humans, the circadian regulation of the LH surge is less rigidly phase-locked to the light cycle than in nocturnal rodents, and the consequences of shift work on ovulation timing are correspondingly harder to measure directly. Nevertheless, diurnal variation in LH pulse frequency and amplitude has been documented in normally cycling women, and this variation is attenuated in women with chronic sleep disruption and irregular work schedules [54]. Mahoney catalogued additional associations between circadian desynchrony and reproductive outcomes, including elevated rates of dysmenorrhea, endometriosis diagnosis, and breast cancer risk in long-term shift workers suggesting that the reproductive consequences of circadian misalignment extend beyond the ovulatory cycle to broader gynecological health [8]. The increased endometriosis prevalence in shift workers is particularly noteworthy given the documented role of clock genes in regulating inflammatory pathways that influence endometrial cell survival and peritoneal implantation, though the mechanistic connection has not been directly established.

The animal data from Summa et al. provide the clearest experimental evidence that circadian disruption per se rather than the confounders that accompany shift work in human populations is sufficient to impair reproductive outcomes [9]. Weekly 12-h phase shifts of the light-dark cycle in pregnant mice produced a significant increase in fetal reabsorption rate compared to unperturbed controls, with the effect size proportional to the number of phase shifts experienced. Importantly, the fetal reabsorption phenotype was not explained by differences in progesterone levels, implying that the mechanism involved implantation or early placental function rather than luteal steroidogenic insufficiency alone [9]. Whether a similar sensitization of early pregnancy to circadian disruption exists in humans and whether it contributes to the elevated miscarriage rates reported in shift workers remains an open question, but the animal data provide a plausible mechanistic framework for the epidemiological associations.

For women undergoing IVF specifically, the relevance of shift work extends beyond ovulation timing. Ovarian stimulation bypasses the natural LH surge, replacing it with a precisely timed trigger injection, which might be expected to eliminate the ovulation timing vulnerability identified in shift workers. However, the follicular clock-dependent processes that operate downstream of the trigger including cumulus expansion, final oocyte maturation, cortical granule redistribution, and the metabolic reprogramming required for meiotic completion would be expected to remain subject to intrinsic granulosa and cumulus cell BMAL1-dependent clock regulation (Section 3.1), based on paracrine signaling mechanisms characterized in mouse models [55], and are therefore hypothesized, but not yet demonstrated, to be impaired in women with chronic circadian misalignment regardless of how precisely the trigger is timed. Whether shift working status independently predicts IVF outcomes after controlling for ovarian reserve and age has not been examined in adequately powered prospective studies a gap that is straightforward to address and clinically relevant given the substantial proportion of reproductive-age women in shift-working occupations.

### 5.3. Sleep Quality and IVF Outcomes

Sleep disturbance and circadian disruption are related but distinct phenomena. Circadian misalignment can occur with normal sleep duration if sleep is scheduled at the wrong biological time, while sleep deprivation can occur without circadian disruption if it is acute and randomly timed. In the context of IVF, both are relevant, but sleep quality has received more direct clinical study because it is measurable with validated instruments in outpatient populations without requiring actigraphy or melatonin profiling [56].

Pamfilio et al. reviewed the available evidence linking sleep quality to IVF outcomes, concluding that sleep habits and disturbances influence clinical pregnancy and live birth rates through several mechanisms: HPA axis dysregulation leading to elevated cortisol, which suppresses GnRH pulsatility and reduces LH pulse amplitude [10]. Altered prolactin secretion, which is normally concentrated in the early morning sleep period and plays a role in corpus luteum support; disrupted growth hormone release, which contributes to granulosa cell proliferation and follicle responsiveness to FSH and immune activation from sleep deprivation, which raises endometrial NK cell activity and may reduce implantation tolerance [10]. These mechanisms are not mutually exclusive and likely operate simultaneously in women with significant sleep disturbance during an IVF cycle.

The most direct clinical evidence comes from Reschini et al., who prospectively enrolled 263 women undergoing IVF and administered the Pittsburgh Sleep Quality Index (PSQI) before the start of stimulation [57]. Women with a PSQI score above 5 indicating poor sleep quality had significantly lower odds of clinical pregnancy than good sleepers after adjusting for age, BMI, ovarian reserve, and number of embryos transferred (adjusted OR 0.50; 95% CI 0.26–0.94; *p* = 0.03). The magnitude of this association is comparable to that of established modifiable risk factors in IVF such as smoking and obesity, yet sleep quality is not routinely assessed in pre-IVF consultations. The study was single-center and relied on self-reported sleep assessment rather than objective actigraphy, and the PSQI captures habitual sleep quality over the preceding month rather than sleep specifically during the stimulation cycle limitations that the authors acknowledged and that reduce the precision of the estimate without undermining the direction of the finding.

What remains unclear from the existing clinical literature is whether the association between poor sleep and IVF failure operates through the HPA and hormonal mechanisms described above, through direct impairment of granulosa-cell BMAL1-dependent clock function and the downstream deficits in steroidogenesis and oocyte quality reviewed in earlier sections, or through both pathways simultaneously. Disentangling these mechanisms would require studies that combine objective sleep monitoring with follicular fluid sampling for clock gene expression and oxidative stress markers a design that is feasible within existing IVF programs and would substantially advance understanding of how sleep disturbance translates into follicular pathology at the molecular level. The clinical contexts in which circadian disruption impairs female reproductive outcomes, the proposed mechanisms, and the strength of available evidence are outlined in Table 2.

## 6. Melatonin, Obesity, and the Disrupted Clock in ART

### 6.1. Melatonin as a Circadian Effector in the Follicle

Melatonin is synthesized in the pineal gland from serotonin through a two-step enzymatic reaction: arylalkylamine N-acetyltransferase (AANAT) converts serotonin to N-acetylserotonin, which is then methylated by hydroxyindole-O-methyltransferase (HIOMT) to produce melatonin [60]. Both enzymes are transcriptionally regulated by the BMAL1-CLOCK heterodimer through E-box elements in their promoters, making melatonin synthesis a direct clock output rather than a passive correlate of darkness [4,59]. AANAT activity is the rate-limiting step and shows a nocturnal surge of 10–100-fold above daytime levels, driving the characteristic rise in circulating melatonin that begins 2–3 h before habitual sleep onset and peaks between 2 and 4 AM. This rhythm is suppressed acutely by light exposure at wavelengths below 530 nm blue-spectrum light through a retinohypothalamic tract projection to the SCN that inhibits sympathetic drive to the pineal gland within minutes. Chronic light-at-night exposure, as experienced by shift workers and heavy screen users, progressively attenuates AANAT induction, reducing both the amplitude and the duration of the nocturnal melatonin peak [7,8].

Within the ovary, melatonin is not simply a circulating hormone that diffuses passively into follicular fluid. Granulosa cells express both melatonin receptors MT1 (MTNR1A) and MT2 (MTNR1B), which are G-protein coupled receptors that signal through Gαi to reduce cAMP accumulation and activate ERK1/2 and AKT pathways [59]. MT1 and MT2 show differential expression across follicular development: MT1 predominates in small antral follicles, while MT2 expression rises in preovulatory follicles coinciding with the period of maximal melatonin accumulation in follicular fluid. Beyond receptor-mediated signaling, melatonin acts as a direct free radical scavenger through a receptor-independent mechanism its indole ring system donates electrons to neutralize hydroxyl radicals, peroxynitrite, and singlet oxygen with rate constants that exceed those of classical antioxidants including vitamin E and glutathione on a molar basis [31,59]. The melatonin molecule itself is oxidized in this process to cyclic 3-hydroxymelatonin, which retains antioxidant activity and can be further oxidized to AFMK and AMK metabolites that also scavenge ROS and upregulate antioxidant enzymes including SOD, catalase, and GPx. This cascading antioxidant effect means that a single melatonin molecule generates a chain of protective metabolites rather than being consumed in a single scavenging reaction [61].

Follicular fluid melatonin concentrations in normally cycling women exceed those in simultaneously sampled serum, with ratios ranging from 1.5 to 3-fold depending on follicle size and time of oocyte retrieval. This accumulation implies active uptake or local synthesis within the follicle rather than simple equilibration with plasma [62]. There is evidence for the latter: granulosa cells express AANAT and HIOMT mRNA, and their expression is inducible by FSH stimulation, suggesting that the follicle contributes to its own melatonin supply independently of the pineal gland. If confirmed at the protein level with functional enzyme activity, this would position the follicular melatonin system as a locally regulated antioxidant mechanism that supplements but does not entirely depend on systemic melatonin input with implications for how shift work-related melatonin suppression translates into follicular oxidative stress [63]. A woman whose pineal melatonin output is chronically attenuated by night shift work may have some compensatory local synthesis in her follicles, but whether this compensation is quantitatively adequate to maintain the antioxidant environment required for normal oocyte maturation is unknown.

At the molecular level, melatonin receptor activation in granulosa cells produces effects that extend well beyond acute cAMP suppression. MT1/MT2 signaling activates the PI3K-AKT pathway, which phosphorylates and inactivates the pro-apoptotic protein BAD, stabilizes mitochondrial membrane potential by preventing VDAC1 opening, and promotes the nuclear exclusion of FOXO3a thereby suppressing the FOXO3a-driven transcription of pro-apoptotic BIM and FASL effects demonstrated in other MT1/MT2-expressing reproductive tissues such as testicular Leydig cells [64] and presumed, but not yet directly confirmed, to operate similarly in granulosa cells. Simultaneously, AKT phosphorylates and activates eNOS, generating low-level nitric oxide that acts as a vasodilator in the follicular wall and a signaling molecule that modulates steroidogenesis. ERK1/2 activation downstream of melatonin receptors promotes granulosa cell proliferation and upregulates CYP19A1 expression, directly enhancing aromatase activity and estradiol output from the preovulatory follicle [65]. These receptor-mediated effects operate in parallel with the direct antioxidant chemistry described above, making melatonin’s action in the follicle genuinely pleiotropic rather than reducible to a single mechanism.

Hu et al. synthesized the clinical evidence for melatonin supplementation in ART in a systematic review and meta-analysis of ten randomized controlled trials [59]. The pooled analysis showed a significant increase in clinical pregnancy rate with melatonin supplementation (OR 1.65; 95% CI 1.14–2.38), accompanied by increases in the number of oocytes retrieved, the proportion of mature MII oocytes, and the number of good-quality embryos on day 3. Follicular fluid oxidative stress markers including 8-hydroxy-2′-deoxyguanosine, a marker of oxidative DNA damage were significantly lower in melatonin-supplemented patients, directly linking the clinical benefit to the antioxidant mechanism. The trials included in the meta-analysis used doses ranging from 1 to 9 mg per day administered from the start of stimulation, and no serious adverse events were reported across any trial. However, the included populations were heterogeneous spanning PCOS, diminished ovarian reserve, and unexplained infertility and no trial was powered to detect a significant difference in live birth rate, which remains the outcome of primary clinical importance. The mechanistic coherence of the finding is stronger than the statistical precision of the estimate, and adequately powered trials in well-defined patient subgroups are needed before supplementation can be formally recommended.

### 6.2. Obesity, NF-κB, and BMAL1 Dysfunction

Obesity impairs female fertility through multiple converging mechanisms: hyperinsulinemia drives ovarian androgen excess, hyperleptinemia suppresses GnRH pulsatility, elevated free fatty acids induce granulosa cell lipotoxicity, and chronic low-grade inflammation characterized by elevated circulating IL-6, TNF-α, and CRP disrupts the intraovarian cytokine environment required for normal folliculogenesis [15,58]. Each of these mechanisms has been documented independently, but they share a common molecular denominator that has received less attention in the reproductive medicine literature: the disruption of BMAL1 function in metabolically active tissues, including the ovary itself.

The central mechanistic insight comes from Maury et al., who performed genome-wide ChIP-seq for BMAL1 and NF-κB (p65 subunit) in omental adipose tissue from obese and lean subjects undergoing bariatric or elective abdominal surgery [15]. In lean subjects, BMAL1 occupied approximately 3500 genomic loci in a circadian phase-dependent manner, with enrichment at E-box elements in the promoters of metabolic and mitochondrial genes. In obese subjects, this binding pattern was profoundly altered: BMAL1 occupancy was reduced at thousands of its canonical sites and gained at others, with the net effect of redirecting BMAL1 transcriptional activity away from metabolic homeostasis genes and toward inflammatory loci. The mechanism was NF-κB-dependent: in obesity, constitutive NF-κB activation driven by saturated fatty acid-TLR4 signaling and adipose tissue macrophage infiltration generates p65 subunit occupancy at genomic sites that overlap with BMAL1 E-box targets. The two transcription factors compete for a shared pool of co-activators including CBP/p300, and under conditions of sustained NF-κB activation, BMAL1 is displaced from its target sites by competitive exclusion rather than direct protein-protein inhibition [15]. Pharmacological inhibition of IKKβ, the kinase that activates NF-κB, restored BMAL1 genomic occupancy and corrected the circadian period in adipocyte precursors from obese subjects, identifying a specific and potentially druggable point of intervention.

Whether this NF-κB-BMAL1 competitive displacement operates in human granulosa cells under conditions of obesity has not been directly tested. The conditions that drive it in adipose tissue sustained TLR4 activation by saturated fatty acids, macrophage-derived cytokine exposure, and constitutive IKKβ activity are all present in the ovarian follicular environment of obese women, where elevated free fatty acid concentrations in follicular fluid, increased macrophage infiltration of the ovarian stroma, and higher intraovarian IL-6 and TNF-α levels have each been documented [15,58]. The mechanistic parallel is therefore plausible and deserves direct investigation in human granulosa cells, particularly given that the downstream consequences of BMAL1 displacement reduced StAR expression, impaired mitochondrial biogenesis, attenuated NRF2-dependent antioxidant defense map directly onto the follicular pathology observed in obese IVF patients.

Schrader et al. provided clinical evidence for the bidirectional relationship between obesity and clock dysfunction at the systemic level. In adipose tissue biopsies from obese and lean women, expression of CLOCK, BMAL1, PER1, CRY2, and REVERBA was significantly altered in the obese group, with the pattern of changes reflecting both reduced clock amplitude and phase disruption rather than uniform downregulation of all clock components [58]. This selective pattern with REVERBA showing the most pronounced reduction is mechanistically significant: REVERBA normally suppresses NF-κB target gene expression by competing with p65 for co-repressor binding, so its loss in obese adipose tissue would be expected to further disinhibit NF-κB signaling, creating a self-reinforcing cycle in which obesity reduces REVERBA, NF-κB activity rises, BMAL1 is displaced from its targets, metabolic gene expression deteriorates, and adipose dysfunction worsens. This feedforward loop has been proposed as a mechanism by which obesity becomes self-perpetuating at the epigenetic and transcriptional level, and its operation within the ovarian follicular environment would predict a similarly self-reinforcing deterioration of follicle quality with increasing adiposity and duration of obesity.

The clinical implication is that weight loss prior to IVF, already advisable on the basis of its effects on insulin resistance, androgen excess, and ovulatory function, may carry an additional benefit through partial restoration of ovarian BMAL1 function. In rodent models of diet-induced obesity, caloric restriction restores circadian gene expression amplitude in metabolic tissues within weeks, and this restoration precedes and predicts the recovery of metabolic parameters. Whether the same applies to human granulosa cells, and whether the timescale of clock restoration is compatible with pre-IVF weight loss programs of 3–6 months duration, has not been studied. It is a tractable question that could be addressed by measuring granulosa cell clock gene expression at oocyte retrieval in women who have undergone structured pre-IVF weight loss programs compared to those who have not a study design that requires no additional intervention beyond what is already collected in standard IVF practice.

## 7. Discussion

The sections above have built a molecular case for BMAL1 as a determinant of oocyte quality and IVF outcomes across steroidogenesis, redox balance, mitochondrial function, and meiotic integrity, and have described the clinical contexts in which this regulatory network breaks down. What the individual sections could not do is examine how these mechanisms interact, where the existing evidence is genuinely strong versus where it is inferred, and what the collective findings mean for clinical practice.

Liu et al. showed that selective BMAL1 loss in steroidogenic cells causes implantation failure through StAR-dependent progesterone deficiency [16]. Wang et al. demonstrated that the same deletion activates PI3K/NF-κB in mouse granulosa cells, adding an inflammatory component to the steroidogenic deficit [28]. Maury et al. then showed in human omental adipose tissue that constitutive NF-κB activation under obese conditions physically displaces BMAL1 from thousands of genomic target sites through competition for CBP/p300 co-activator [15]. Schrader et al. confirmed in clinical samples that obesity reduces REVERBA expression alongside BMAL1, and since REVERBA normally suppresses NF-κB target genes, its loss disinhibits inflammatory signaling further [58]. The result is a self-reinforcing loop: obesity activates NF-κB, which displaces BMAL1, which fails to drive REVERBA, which permits more NF-κB activity.

For the obese woman undergoing IVF, this loop has a concrete consequence: her granulosa cells are operating with a BMAL1 cistrome reorganized by chronic inflammatory signaling, reducing transcriptional drive to StAR, CYP19A1, NRF2, and PGC-1α simultaneously. Weight loss before IVF already advisable on metabolic and endocrine grounds may carry an additional benefit through partial restoration of BMAL1 genomic occupancy as NF-κB activity falls. In rodent models of diet-induced obesity, caloric restriction restores circadian gene expression amplitude in metabolic tissues within weeks and precedes recovery of metabolic parameters [58]. Whether the same occurs in human granulosa cells over a timescale compatible with pre-IVF weight loss programs has not been tested, but the question is answerable with existing IVF biobank infrastructure.

Brzezinski et al. documented broad attenuation of granulosa cell clock gene expression in women aged 39–45 compared to 18–33, with PER1 and CLOCK reaching statistical significance. The mechanism they did not investigate is NAD+ decline [48]. Miao et al. showed that NAD+ levels fall substantially in aged mouse ovaries and that NMN supplementation restoring those levels improved ovulation rate, meiotic competency, and euploidy by maintaining normal spindle and chromosome structure and the dynamics of the cortical granule component ovastacin [66]. Bertoldo et al. similarly demonstrated that NAD+ repletion rescues female fertility during reproductive aging in mice, with improvements in oocyte quality, fertilization rate, and embryo development attributable to restoration of mitochondrial function and reduction in meiotic spindle defects [67]. The mechanistic bridge between these NAD+ findings and the Brzezinski et al. clock gene data is SIRT1: as NAD+ falls with age, SIRT1 activity declines proportionally, BMAL1 deacetylation decreases, and clock amplitude attenuates with downstream effects on StAR, NRF2, and mitochondrial biogenesis that compound each other [48]. This places the NAD+-SIRT1-BMAL1 axis as the likely molecular mechanism connecting age-related granulosa cell clock attenuation to the follicular deficits that characterize older IVF patients, though this specific causal chain has not been directly tested in human granulosa cells.

Amano et al. made an observation that fundamentally reframes where BMAL1’s influence on oocyte quality operates [68]. They demonstrated that while clock gene transcripts are detectable in germinal vesicle oocytes, they do not oscillate in mouse preimplantation embryos, implying that the transcriptional circadian clock stops at or around meiotic resumption. Stanton et al. confirmed this across four mammalian species human, cattle, pig, and mouse using publicly available RNAseq datasets, showing that CLOCK, ARNTL, PER1, PER2, CRY1, and CRY2 transcript abundance declines progressively from the oocyte to the blastocyst stage in all species, and concluding that absence of a functional molecular clock in the preimplantation embryo is a conserved mammalian characteristic [69]. Global transcriptional silencing during germinal vesicle breakdown removes the BMAL1-driven feedback loop from the oocyte at precisely the stage when meiotic spindle assembly and chromosomal segregation place the highest demands on metabolic and redox precision.

The implication is direct: BMAL1’s influence on oocyte quality is mediated primarily through granulosa cells rather than through an oocyte-intrinsic clock. The granulosa cell clock controls the paracrine and metabolic environment on which the oocyte depends steroidogenic output, glutathione precursor transfer via connexin-43 gap junctions, pyruvate supply from cumulus cell glycolysis, and BMP and EGF signaling coordinating cumulus expansion with meiotic progression. When granulosa cell BMAL1 function fails, this architecture deteriorates at multiple levels simultaneously, and the oocyte cannot compensate. Amano et al. added a further layer by showing that CRY1 knockdown in germinal vesicle oocytes reduced meiotic maturation ability without affecting transcription of canonical clock targets including Wee1, Per1, and Per2 [68]. This indicates that CRY1 performs a clock-independent function in meiosis. The downstream meiotic targets of this CRY1 interaction have not been identified, but the finding complicates the interpretation of clock gene knockout phenotypes: not all reproductive deficits in Bmal1 or Cry1 mutants necessarily reflect disrupted circadian timekeeping. Some reflect the non-circadian biological roles that clock proteins have acquired in germ cell biology.

Hu et al. demonstrated in their meta-analysis that melatonin supplementation during ART cycles increases clinical pregnancy rates (OR 1.65; 95% CI 1.14–2.38) and reduces follicular fluid oxidative stress markers [59]. The mechanistic basis is dual: MT1/MT2 receptor activation in granulosa cells engages PI3K-AKT, stabilizes mitochondrial membrane potential through BAD phosphorylation and VDAC1 regulation, and upregulates CYP19A1 via ERK1/2; receptor-independent antioxidant chemistry neutralizes hydroxyl radicals and peroxynitrite through the indole ring system and generates secondary antioxidant metabolites cyclic 3-hydroxymelatonin, AFMK, and AMK that further upregulate SOD, catalase, and GPx. Neither mechanism requires a functioning granulosa cell clock, which is why melatonin supplementation can in principle benefit patients regardless of their degree of circadian disruption.

What Hu et al. cannot answer is whether the clinical benefit operates through antioxidant protection, clock synchronization, or both [59]. Melatonin is not only a scavenger it is the primary entrainment signal for peripheral circadian oscillators including the granulosa cell clock, and its suppression by shift work or light-at-night exposure documented by Murphy and Sellix and Mahoney removes both the antioxidant protection and the synchronizing signal simultaneously [5,8]. Exogenous supplementation at a fixed evening dose could restore both. None of the trials in the Hu et al. meta-analysis measured dim-light melatonin onset, actigraphy-derived circadian alignment, or granulosa cell clock gene expression [59]. The antioxidant and circadian effects therefore remain conflated in the existing clinical data, and trials stratifying patients by circadian alignment status before randomization are needed to distinguish them.

Murphy and Sellix, Mahoney, and Summa et al. collectively document that circadian disruption impairs female reproductive outcomes through disrupted LH surge timing, irregular GnRH pulsatility, and increased fetal reabsorption rates [5,8,9,53]. The LH surge mechanism is the most vulnerable point in natural conception and IVF bypasses it entirely with a pharmacological trigger. This might suggest that shift workers are less disadvantaged in IVF than in natural conception. Summa et al. complicate this [9]. Their fetal reabsorption phenotype in light-cycle-disrupted mice occurred independently of progesterone levels, implicating implantation or early placentation as the affected process rather than ovulation timing. Zhou et al. provide the molecular mechanism: BMAL1/REV-ERB signaling controls BMP2, BMP4, GDF10, and GDF15 expression in uterine endometrial stromal cells during the implantation window by driving REV-ERBα occupancy at RORE motifs in their promoters, synchronizing decidualization with circadian timing [70]. Circadian misalignment in the endometrium independently of any ovarian effect could impair trophoblast invasion even when a euploid embryo is transferred at the correct developmental stage. In current IVF practice, embryo transfer timing is determined by endometrial preparation protocol and embryo developmental stage, not by the patient’s circadian phase. Whether aligning transfer with the phase of maximal endometrial BMAL1 activity could improve implantation rates is mechanistically plausible but has no clinical evidence base [71,72].

Reschini et al. found that poor sleep quality (PSQI > 5) independently halved the odds of clinical pregnancy in 263 IVF patients (adjusted OR 0.50; 95% CI 0.26–0.94; *p* = 0.03) after controlling for age, BMI, ovarian reserve, and embryo number [57]. Pamfilio et al. proposed several non-exclusive mechanisms: HPA-driven cortisol elevation suppressing GnRH pulsatility, disrupted prolactin secretion impairing corpus luteum support, impaired growth hormone release reducing granulosa cell FSH responsiveness, and immune activation elevating endometrial NK cell activity [10]. Neither Reschini et al. nor Pamfilio et al. can determine whether the association operates through these hormonal and immune mechanisms, through direct impairment of granulosa cell BMAL1 function, or through both simultaneously [10,57]. Reschini et al. measured sleep quality at baseline with a self-reported questionnaire, collected no follicular fluid, and had no granulosa cell molecular data [57]. The critical missing study combines objective circadian alignment assessment actigraphy plus dim-light melatonin onset with granulosa cell clock gene profiling at oocyte retrieval and linked IVF outcome data. Without it, the sleep-IVF association rests on epidemiology rather than mechanism, reasonable to act on clinically but not yet precision medicine.

Voros et al. proposed that the SIRT1-NRF2 axis functions as a critical anti-ferroptotic system in granulosa cells through GPX4 maintenance and SLC7A11-mediated cystine import for glutathione synthesis, and that disruption of this axis drives granulosa cell death through iron-dependent lipid peroxide accumulation alongside conventional apoptosis [30]. The connection to BMAL1 runs through SIRT1 and NRF2 directly. When BMAL1 fails to sustain SIRT1 expression rhythmically, NRF2 stability falls, GPX4 transcription declines, and the glutathione pool is depleted creating conditions in which lipid hydroperoxide accumulation escapes neutralization and triggers ferroptotic death in granulosa cells. This pathway operates in parallel with, and additively with, the apoptotic pathway driven by mitochondrial permeability transition pore opening described in Section 4.2, meaning that BMAL1 dysfunction sensitizes granulosa cells to two distinct regulated death programs simultaneously.

Whether ferroptosis contributes to granulosa cell loss in women with circadian disruption has not been directly tested. It is measurable with established markers acyl-4-hydroxynonenal protein adducts, ferroptosis-specific lipid peroxide species quantified by lipidomics, and GPX4 protein levels by immunofluorescence in follicular fluid or granulosa cells collected at retrieval. Given that the follicular fluid proteome and lipidome are already being characterized in IVF research programs, adding ferroptosis markers to existing collection protocols would require minimal additional infrastructure. The question of whether clock-disrupted patients show elevated follicular ferroptosis markers, and whether these correlate with oocyte quality metrics, is therefore tractable in the near term.

Three claims warrant explicit qualification. First, timing IVF procedures according to the patient’s circadian phase has no clinical evidence base. The granulosa cell clock regulates gene expression in a phase-dependent manner, and steroidogenic gene transcription peaks at specific circadian phases in rodent models. But no clinical study has tested whether oocyte retrieval or embryo transfer at one time of day produces better outcomes than another, and the pharmacological override of the LH surge in IVF already disrupts the natural temporal relationship between trigger administration and follicular clock phase. The mechanistic rationale exists; the clinical data do not.

Second, routine melatonin supplementation for all IVF patients is not supported by the available evidence. Hu et al. showed benefit across heterogeneous populations, but none of the included trials selected patients on the basis of documented circadian disruption, melatonin deficiency, or elevated follicular oxidative stress [59]. A woman with normal nocturnal melatonin output and intact sleep architecture who receives exogenous melatonin supplementation is not correcting a deficit she is receiving a pharmacological dose of a hormone whose follicular effects at supraphysiological concentrations are not fully characterized. The subgroup most likely to benefit women with documented circadian misalignment, suppressed dim-light melatonin onset, or elevated follicular fluid oxidative stress markers has not been specifically studied.

Third, clinical NAD+ precursor supplementation recommendations for IVF patients are premature. Miao et al. and Bertoldo et al. demonstrated compelling oocyte quality benefits in aged rodents, and the mechanism NAD+ restoration driving SIRT1 reactivation, BMAL1 stabilization, and downstream mitochondrial and antioxidant recovery is coherent and mechanistically connected to the ovarian clock biology reviewed throughout this paper [66,67]. But rodent NMN doses of 200–500 mg/kg/day do not translate directly to human equivalents without pharmacokinetic data specific to the ovarian compartment, and no clinical trial has examined NAD+ precursor supplementation with oocyte quality or live birth rate as primary endpoints. The biology is compelling enough to justify designing such a trial, it is not compelling enough to justify clinical recommendations in advance of one. The mechanistic hypotheses generated by this review and the study designs needed to test them clinically are outlined in Table 3.

### Strengths and Limitations of the Clinical Evidence

The clinical evidence underlying this review is asymmetric in quality relative to the molecular mechanisms described in Section 3 and Section 4. Most human associations rest on single studies whose designs constrain causal interpretation. The sleep-IVF association (Reschini et al., adjusted OR 0.50) comes from a single-center cohort using self-reported, habitual sleep quality rather than cycle-specific or objective measurement, and no follicular fluid or granulosa cell data were collected to test a molecular mechanism directly (Section 5.3). The melatonin meta-analysis (Hu et al., OR 1.65) pools heterogeneous patient populations without stratifying by documented circadian disruption, and no included trial was powered to detect a live birth difference (Section 6.1). The granulosa cell clock-aging association (Brzezinski et al.) is a cross-sectional comparison between two small age groups (*n* = 10 total: 5 women aged 18–33, 5 aged 39–45) rather than a longitudinal study, and its consequences for oocyte or embryo outcomes have not been measured in the same patients (Section 5.1). The BMAL1-steroidogenesis correlation in human granulosa cells (Kawamura et al.) is observational at the patient level; causality is established only in the accompanying cell-line experiments, not in patients (Section 3.3). None of the shift-work reproductive literature reviewed here derives from a randomized design, for the practical reason that occupational exposure cannot be randomized; residual confounding by lifestyle and socioeconomic factors cannot be fully excluded despite adjustment in the cited cohort studies (Section 5.2). The associations reported throughout this review, including the sleep, melatonin, aging, and shift-work findings above, should be read as such: none of the human clinical data reviewed here establish causality, and no randomized controlled trial has yet tested whether correcting circadian disruption improves IVF outcomes. Table 3 outlines the specific unanswered questions and study designs needed to close this gap.

The most informative immediate step would be a prospective observational study combining actigraphy-validated circadian alignment assessment with granulosa cell clock gene profiling at oocyte retrieval, linked to fertilization rate, embryo morphokinetics, euploid embryo rate, and live birth. This study requires no additional intervention beyond what standard IVF programs already collect, and it would establish whether the magnitude of the granulosa cell clock-IVF outcome relationship in humans justifies the clinical trials that the molecular data suggest. Without that anchor in human data, the field risks building an increasingly elaborate mechanistic superstructure on a clinical foundation that has not yet been laid.

Beyond this, four specific trials are scientifically justified by the evidence reviewed here: a melatonin supplementation RCT stratified by circadian alignment status; an NMN supplementation RCT in women over 38 with granulosa cell NAD+ and BMAL1 expression as secondary endpoints; a prospective cohort study of shift workers undergoing IVF with endometrial clock gene profiling at transfer and a weight loss intervention study measuring granulosa cell BMAL1 cistrome changes before and after structured pre-IVF weight loss in obese patients. Each addresses a specific mechanistic hypothesis generated by the reviewed evidence and each is feasible within existing reproductive medicine infrastructure.

## 8. Conclusions

BMAL1-driven circadian rhythmicity is not peripheral to ovarian biology; it is embedded in the core processes that determine follicle quality, steroid output, and embryo developmental potential. Its disruption, through whatever mechanism, converges on the same endpoints: insufficient progesterone, elevated follicular oxidative stress, impaired mitochondrial support for the oocyte, and increased susceptibility to meiotic error and granulosa cell death.

The evidence reviewed here is mechanistically coherent but clinically underdeveloped. The molecular case built from conditional knockout models, human granulosa cell studies, redox biology, and metabolic aging research is strong enough to justify reframing circadian health as a modifiable variable in reproductive medicine, alongside the established factors of weight, smoking, and ovarian reserve. It is not yet strong enough to support specific supplementation protocols or procedural timing recommendations.

What is justified now is modest and practical. Sleep quality assessment before IVF is a reasonable, low-risk addition to pre-cycle counseling; actively optimizing sleep or circadian alignment to improve IVF outcomes remains a promising area for investigation, not yet an established intervention. Obese patients should be counseled that weight loss may restore ovarian clock function in addition to its established metabolic benefits. Melatonin supplementation likewise remains a promising area for investigation in patients with documented circadian disruption; it is not yet an intervention ready for routine IVF practice. And the NAD+-SIRT1-BMAL1 axis, which connects reproductive aging to clock deterioration through a pharmacologically targetable mechanism, deserves prospective clinical investigation. The broader reframing this review argues for is that the follicular microenvironment is not static between stimulation cycles it is temporally organized, clock-dependent, and sensitive to the circadian state of the patient at the time of follicle development. Treating it as such, and designing studies that measure ovarian clock function alongside conventional IVF outcome parameters, is the most productive next step the field can take.

## Figures and Tables

**Table 1:** BMAL1-regulated steroidogenic genes in ovarian cells: experimental evidence and translational relevance.

Gene	Encoded Protein	Cell Type	Effect of BMAL1 Loss	Model	Evidence Source
STAR [16]	Steroidogenic Acute Regulatory Protein	Granulosa, theca, luteal	Marked downregulation; cholesterol transport arrested	SF1-Bmal1^−/−^ mouse	Animal model (mouse)
CYP11A1 [17]	Cholesterol side-chain cleavage enzyme	Granulosa	Reduced expression; pregnenolone synthesis impaired	Human LGC; KGN/HGL5	Human tissue + cell line
HSD3B2 [28]	3β-hydroxysteroid dehydrogenase	Granulosa, theca	Downregulated; progesterone synthesis reduced	Bmal1-KD mouse LFC	Animal model (mouse)
CYP19A1 [17,28]	Aromatase	Granulosa	Reduced expression and 17β-estradiol output	Human LGC; KGN/HGL5	Human tissue + cell line
LHCGR [28]	LH/hCG receptor	Granulosa	Downregulated; LH responsiveness blunted	Bmal1-KD mouse LFC	Animal model (mouse)
ESR2 [17]	Estrogen receptor β	Granulosa	Positive correlation with BMAL1 expression	Human LGC	Human tissue

LGC, luteinized granulosa cells; LFC, luteinized follicle cells; KD, knockdown; knockout. All effects reflect loss-of-function conditions unless stated otherwise. Human data are derived from IVF patients at oocyte retrieval or from established granulosa cell lines (KGN, HGL5).

**Table 2:** Clinical contexts of circadian disruption and their reproductive consequences: mechanisms and evidence base.

Clinical Context	Reproductive Outcome	Proposed Mechanism	Evidence Level	Studies/Participants (As Reported in the Cited Sources)
Night shift work/rotating schedules [5,8,53]	Menstrual irregularity, delayed conception, elevated miscarriage rate	Disrupted LH surge timing; altered GnRH pulsatility; ovarian clock gene desynchronization	Epidemiological + animal	Narrative synthesis of multiple human cohort/epidemiological studies and rodent models (reviews; no single pooled sample size reported)
Environmental light-cycle perturbation [9]	Increased fetal reabsorption; impaired pregnancy maintenance	Circadian disruption of implantation-related processes independent of progesterone levels	Animal (mouse)	48 pregnant mice across 3 groups (12 control, 18 phase-delay, 18 phase-advance)
Poor sleep quality (PSQI > 5) [57]	Reduced clinical pregnancy rate in IVF (adjusted OR 0.50)	HPA axis dysregulation; disrupted prolactin and GH secretion; elevated endometrial NK cell activity	Prospective clinical cohort	263 women (single-center prospective cohort)
Aging (39–45 vs. 18–33 years) [48]	Reduced oocyte competence; impaired steroidogenic support	Attenuation of granulosa cell clock amplitude; NAD+-SIRT1-BMAL1 axis decline	Human granulosa cell study	10 women total (5 aged 18–33; 5 aged 39–45)
Obesity/metabolic inflammation [14,58]	Impaired folliculogenesis; reduced IVF success	NF-κB-BMAL1 competitive displacement; REVERBA suppression; adipokine-driven intraovarian inflammation	Human adipose tissue; clinical cohort	8 obese vs. 8 lean subjects (Maury et al. ChIP-seq/expression cohort); Schrader et al. is a review additionally citing a cohort of 21 lean and 28 obese women
Melatonin deficiency (shift work, light-at-night) [59]	Elevated follicular oxidative stress; reduced oocyte maturation	Loss of MT1/MT2-mediated mitochondrial protection; impaired granulosa cell peripheral clock entrainment	RCT meta-analysis	10 randomized controlled trials (pooled meta-analysis)

PSQI, Pittsburgh Sleep Quality Index; OR, odds ratio; GH, growth hormone; HPA, hypothalamic–pituitary–adrenal; NK, natural killer; RCT, randomized controlled trial. Evidence levels: epidemiological = human observational data without intervention; animal = rodent experimental models; prospective clinical cohort = human longitudinal data with defined outcome measures; RCT meta-analysis = pooled data from randomized controlled trials.

**Table 3:** Unanswered clinical questions arising from the reviewed evidence and proposed study designs.

Unanswered Question	Current Evidence Status	Proposed Study Design	Section
Does timing oocyte retrieval or embryo transfer to the patient’s circadian/follicular clock phase improve IVF outcomes?	Mechanistic rationale only (rodent phase-dependent steroidogenic gene transcription); no clinical study has tested procedure timing against outcomes	Prospective observational study combining actigraphy-validated circadian alignment with granulosa cell clock gene profiling at retrieval, linked to fertilization rate, embryo morphokinetics, euploid rate, and live birth	Section 7
Does melatonin supplementation benefit only patients with documented circadian disruption, rather than all IVF patients?	Meta-analysis shows benefit across heterogeneous, unselected populations (OR 1.65); no trial stratified by circadian alignment or powered for live birth	Melatonin supplementation RCT stratified by circadian alignment status	Section 6.1 and Section 7
Does NAD+ precursor supplementation improve oocyte quality or IVF outcomes in older women through clock restoration?	Benefit demonstrated in aged rodent models; no human trial; rodent doses not yet translated to human pharmacokinetics	NMN supplementation RCT in women over 38, with granulosa cell NAD+ and BMAL1 expression as secondary endpoints	Section 5.1 and Section 7
Does shift-work status independently predict IVF outcomes, and does endometrial clock desynchronization impair implantation?	Epidemiological associations documented for natural-conception outcomes only; no adequately powered prospective IVF study; endometrial clock mechanism inferred from animal data	Prospective cohort study of shift workers undergoing IVF with endometrial clock gene profiling at embryo transfer	Section 5.2 and Section 7
Does pre-IVF weight loss restore granulosa cell BMAL1 function on a clinically relevant timescale?	Restoration demonstrated in rodent models of diet-induced obesity within weeks; not studied in human granulosa cells	Weight-loss intervention study measuring granulosa cell BMAL1 cistrome changes before and after structured pre-IVF weight loss in obese patients	Section 6.2 and Section 7
Does the association between poor sleep quality and lower IVF pregnancy rates operate through hormonal/HPA mechanisms, direct granulosa clock impairment, or both?	Single-center cohort study (adjusted OR 0.50) using self-reported, habitual sleep quality; no follicular fluid or granulosa cell data collected	Study combining objective circadian alignment assessment (actigraphy plus dim-light melatonin onset) with granulosa cell clock gene profiling at retrieval and linked IVF outcome data	Section 5.3 and Section 7

The study designs listed above are proposed avenues for future research generated by the evidence reviewed in this manuscript. They represent research recommendations, not evidence-based clinical protocols, and none has yet been tested prospectively.

## Data Availability

No new data were created or analyzed in this study. Data sharing is not applicable to this article.

## Abbreviations

ART	Assisted Reproductive Technology
BMAL1	Brain and Muscle ARNT-Like Protein 1
CLOCK	Circadian Locomotor Output Cycles Kaput
IVF	In Vitro Fertilization
ROS	Reactive Oxygen Species
NRF2	Nuclear Factor Erythroid 2–Related Factor 2
SIRT1	Sirtuin 1
NF-κB	Nuclear Factor Kappa B
PGC-1α	Peroxisome Proliferator-Activated Receptor Gamma Coactivator 1 Alpha
LH	Luteinizing Hormone
FSH	Follicle-Stimulating Hormone
SCN	Suprachiasmatic Nucleus
HPO	Hypothalamic-Pituitary-Ovarian
MII	Metaphase II
PSQI	Pittsburgh Sleep Quality Index
NAD^+^	Nicotinamide Adenine Dinucleotide
